# HBx Down-Regulated Gld2 Plays a Critical Role in HBV-Related Dysregulation of miR-122

**DOI:** 10.1371/journal.pone.0092998

**Published:** 2014-03-25

**Authors:** Feng Peng, Xinqiang Xiao, Yongfang Jiang, Kaizhong Luo, Yi Tian, Milin Peng, Min Zhang, Yun Xu, Guozhong Gong

**Affiliations:** Department of Infectious Diseases, Second Xiangya Hospital of Central South University, Changsha, Hunan, China; Academia Sinica & National Defense Medical Center, Taiwan

## Abstract

miR-122 is a liver-rich-specific microRNA that plays an important role in hepatic gene expression via post-transcription regulation, and it is potentially associated with the development of hepatocellular carcinoma. It has been confirmed that miR-122 is down-regulated during HBV infection; however, how HBV affects miR-122 is still debated. One research provided evidence that HBx could reduce the miR-122 transcription level, but the other insisted that HBV had no significant effect on miR-122 transcription level but reduce miR-122 level via binding and sequestering endogenous miR-122. It is determinate that Gld2 could increase the specific miRNA stabilization by monoadenylation which was a post-transcription regulation. In this study, we aimed to investigate the mechanism of HBV-induced reduction of miR-122 and examine whether Gld2 is involved in it. According to the results of a microRNA microarray, we found miR-122 was the most down-regulated microRNA in HepG2.2.15 compared to HepG2. As revealed by qRT-PCR and western blotting analyses, both miR-122 and Gld2 levels were reduced in hepatic cell lines with expression of HBV or HBx but not other proteins of HBV, and over-expression of Gld2 could abolish the effect of HBV and HBx on the miR-122 level. What's more, both HBV and HBx have no significant effect on pre-miR-122 levels. And the dual-luciferase assay implicated that HBx could reduce the Gld2 promoter activity but had no significant effect on miR-122 promoter activity. In conclusion, HBx is a critical protein derived from HBV, which regulates miR-122 via down-regulating Gld2.

## Introduction

MiR-122, a liver-specific microRNA (miRNA), constitutes more than 70% of the total miRNAs in adult human liver and regulates gene translation via binding to the 3′ -untranslated region (3′-UTR) of its target messenger RNA (mRNA) [1], [2]. It has been reported that miR-122 is very important for maintaining liver function, such as the regulation of cholesterol and fatty-acid metabolism [3]–[5]. Several miR-122 target genes, which contribute to tumour-genesis, have been identified, such as ADAM10, IGF1R, CCNG1, Bcl-ω, and ADAM17 [6]–[8], and low miR-122 levels are associated with hepatocellular carcinoma (HCC) [9], for example, miR-122 level was frequently reduced in HCC tissue compared to non-tumour tissue, low miR-122 was also correlated with poor prognosis [10], [11], and over-expression of miR-122 inhibited tumour cell growth [12]. Taken together, these findings indicated that miR-122 is functioning as a tumour suppressor gene. Recent studies showed miR-122 not only regulates cellular gene expression, but also plays an important role in virus replication. Hepatitis C virus (HCV) replication was enhanced by miR-122 by interacting with 5′-NCR [13], [14], In contrast, miR-122 was found to inhibit hepatitis B virus (HBV) replication [15]. Furthermore, in chronic HBV infection patients, miR-122 was negatively correlated with intra-hepatic viral load and hepatic necro-inflammation [16]. Until now, there are two different explanations for the mechanism of HBV-related miR-122 down-regulation. One hypothesis is that the highly redundant HBV transcripts are involved in HBV-mediated miR-122 suppression by binding and sequestering endogenous miR-122, and in this process, the transcription level of miR-122 is not affected directly [17]; while the other is that hepatitis B virus X protein (HBx) binds PPARγ (peroxisome proliferator-activated receptor gamma) and results the inhibition of the miR-122 transcription level [18] which is in conflict with the former. Hence, more experimental evidence is needed to explore the mechanism of HBV-related inhibition of miR-122.

Germline development 2 (Gld2, also called PAPD4) is a cytoplasmic poly(A) RNA polymerase that adds successive AMP monomers to the 3′-end of specific RNAs, thereby forming a poly(A) tail, and controls mRNA translation [19]. Gld2 enzymes acquire substrate specificity by interacting with RNA-binding proteins and are recruited to only a subset of mRNAs [20]. Previous studies have mostly focused on the effect of Gld2 on mRNA. Recently, Joel D. et al. demonstrated that Gld2 could increase specific miRNA stabilisation via monoadenylation in human fibroblasts, and specifically, miR-122 could be stabilized by Gld2 [21], [22]. A previous study performed in Japan [23] also demonstrated significantly lower levels of miR-122 in the livers of Gld2-null mice. These two studies indicated that Gld2 could regulate microRNA expression at the post-transcription level.

Here, we studied the effect of HBV on the expression of miR-122 in vitro. Interestingly, we found that HBV reduced miR-122 levels by down-regulating the Gld2 gene, which might represent a new mechanism for regulating the expression of miRNAs by HBV. In addition, we confirmed that the HBx protein plays a critical role in down-regulating Gld2 protein and subsequently results in a reduction in miR-122 levels.

## Materials and Methods

### Plasmid constructions

The Gld2-expressing plasmid was cloned by PCR from human cDNA (the reverse transcription product from human hepatic cell line QSG7701) using the following primers: 5′-CGAAGCTTATGTTCCCAAACTCAATTTT -3′ (sense) and 5′- TAGGGCCCTTATCTTTTCAGGACAGCAG -3′ (antisense). pcDNA3.1 (pcDNA) was used as the expression vector. The expression plasmid was named pGld2.

The expression plasmids for the four proteins of HBV (HBx protein, surface antigen (HBsAg), core protein (HBcAg), and DNA polymerase protein (HBp)) were cloned using PCR from pHBV1.3, which was kindly presented by Doctor Songdong Meng from the Chinese Academy of Sciences (CAS) [17]. The pcDNA3.1 (pcDNA) was also used as the expression vector. The four plasmids were named pHBx, pHBs, pHBc, and pHBp, respectively.

We designed siRNA targeting HBx mRNAs according to the GenScript siRNA Target Finder (https://www.genscript.com/ssl-bin/app/rnai). The sense and antisense oligonucleotides, which constituted the template for generating the siRNAs, were subcloned into the pRNAT-U6.1/Neovector with the U6-RNA promoter between the HindIII and BamHI restriction sites. The last sequences selected for gene silencing in our study were 5′-TTCACCTCTGCACGTTGCA-3′ (sense) and 5′-TGCAAC GTGCAGAGGTGAA-3′ (antisense). The HBx siRNA-expressing plasmids were named psiHBx.

The miR-122 and Gld2 promoter reporter plasmids were cloned using PCR from human genomic DNA with the following primers: miR-122 promoter: 5′-CGATACGCGTGAATGCATGGTTAAC-3′ (sense) and 5′-TGATCTCGAGCCT CCCGTCATTTCT-3′ (antisense); and Gld2 promoter: 5′-GATACGCGTTTT TGAGACAAAGTCTTGCT-3′ (sense) and 5′-GAGCTCGAGACCCCGCCTAT GAGCGCTTT-3′ (antisense). The miR-122 promoter sequences were 661 bps, which contained human miR-122 gene sequences that spanned the region between -496 and +165 bp [24]. The Gld2 promoter sequences in this study were 644 bps, which were the forward sequences of the transcription initiation site (TSS) of the Gld2 gene (between -142 and -785) and were designed based on the published sequence of Gld2 (GenBank accession no. BC026061.1). Both of the promoter sequences were inserted into the MluI and XhoI sites of the pGL3-Basic Luciferase Reporter Vector, which is a firefly luciferase (FL) expression vector. The two promoter reporter plasmids were named p122-luc and pGld2-luc. These plasmids were validated by sequencing.

### Cell culture and transfection

HepG2, HepG2.2.15, Huh-7, and QSG7701 cell lines were cultured with Dulbecco's modified Eagle's medium (DMEM) supplemented with 10% foetal bovine serum (FBS) and 1% penicillin/streptomycin. The cells were maintained in a humidified incubator at 37°C with 5% CO_2_. Twenty-four hours prior to transfection, the cells were seeded into 6-well plates in antibiotic-free growth medium at a density of 3×10^5^ cells/well. After reaching 80% confluency, the cells were transfected with vectors using Lipofectamine 2000 according to the manufacturer's instructions. The growth medium was changed after 4 h, and the total cellular RNA or protein was isolated for the next experiment.

### MicroRNA microarray and real-time PCR

Isolation of the total cellular RNA was performed using TRIzol reagent. Affymetrix microRNA 2.0 array was used to perform the microRNA gene microarray. Complementary DNA was synthesised from total RNA (both mRNA and microRNA) using the TaKaRa One step PrimeScript miRNA cDNA Synthesis Kit (Perfect Real Time) according to the manufacturer's instructions. To quantify the target mRNA or miRNAs, Quantitative real-time polymerase chain reaction (qRT-PCR) was performed using the ABI 7500 Real-Time PCR System with Takara SYBR_Premix Ex TaqTM II (Perfect Real Time) according to the manufacturer's instructions. The forward primers of each target mRNA or miRNA are described as follows: β-actin: 5′-CCAACTGGGACGACAT-3′(sense) and 5′-AGCCTGGATAGC AACG-3′(antisense); HBx: 5′-TCTGTGCCTTCTCATCTGC-3′ (sense) and 5′-TCG GTCGTTGACATTGCTG-3′ (antisense); Gld2: 5′-TTCGTCCGTTAGTGCTG G-3′ (sense) and 5′-GGGATGGAAGGATGGGTT-3′(antisense); U6: 5′-CGCTTCGG CAGCACATATAC -3′(sense) and universal primers provided in the TaKaRa One step PrimeScript miRNA cDNA Synthesis Kit(antisense); miR-122: 5′- TCGCCTGGAGTGTGACAATGG - 3′(sense) and universal primers provided in TaKaRa One step PrimeScript miRNA cDNA Synthesis Kit (antisense); precursor miR-122: 5′-TTAGCAGAGCTGGGAGT-3′ (sense) and 3′-GCCTAGCAGTAGCTATTT-3′ (antisense). The levels of miRNA and mRNA expression were measured using the Ct (threshold cycle) method. The ΔΔCt method for relative quantification of gene expression was used to determine the miRNA or mRNA expression levels. ΔCt was calculated by subtracting the Ct of U6 (for miRNA) or β-actin (for mRNA) RNA from the Ct of the miRNA or mRNA of interest. The ΔΔCt was calculated by subtracting the ΔCt of the reference sample (non-tumour liver tissue) from the ΔCt of each sample. The fold change was determined using the equation 2^-ΔΔCt^.

### Western blotting analyses

Cells were harvested and lysed in 500 μl cell culture lysis reagent according to the protocol provided by the manufacturer. Cell lysates were then clarified by centrifugation, and the protein concentration of each sample was determined using the BCA Protein Assay Kit. Standard western blotting procedures were performed. The primary antibodies used were as follows: mouse monoclonal anti-HBx, goat polyclonal anti-Gld2, and mouse monoclonal anti-β-actin. The secondary antibodies were goat anti-mouse IgG-HRP and donkey anti-goat. Antibody/antigen complexes were detected using the SuperSignal West Pico Chemiluminescent Substrate.

### Dual-luciferase assay

To study the effects of HBV, HBx, HBsAg, HBcAg, and HBp on miR-122 and Gld2 promoter activity, the expression plasmids for these genes or pcDNA, miR-122, or Gld2 promoter-luciferase reporter plasmids and Renilla luciferase (RL) expression vector (PRL-TK) were mixed (4∶0.5∶0.1) and co-transfected into cells cultured in 6-well plates. The cells were split, and the FL/RL activities were measured 24 h after transfection using the Dual-Luciferase Assay Kit.

### Data analysis

All studies were performed in three separate experiments, where triplicate wells were transfected. Data analysis was performed using the two-tailed Student's t-test with pooled variance. The data are expressed as the mean ± SD. A P-value of <0.05 was considered significant. Statistical analysis was performed using SPSS 16.0 software.

## Results

### HBV decreases mature miR-122 level in various hepatocyte cell lines

**Figure 1:**
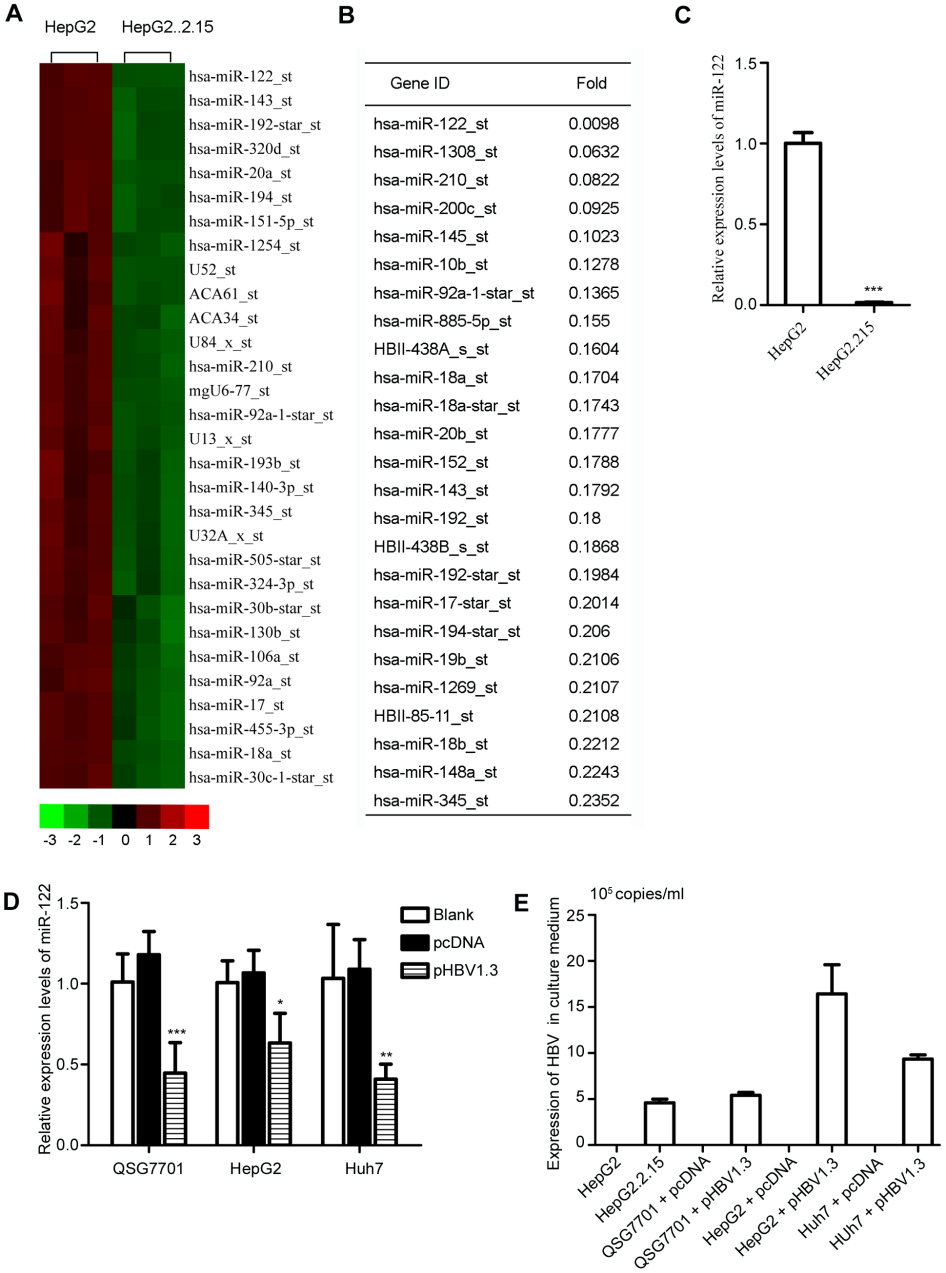
miR-122 is down-regulated by HBV in hepatic cells. (A) miRNA expression heat map depicting differentially expressed miRNAs (p<0.05) in HepG2 and HepG2.2.15 cells. (B) A total of 151 miRNAs were down-regulated in HepG2.2.15 compared to HepG2 cells (p<0.05). (C) qRT-PCR analysis of miR-122 in HepG2 and HepG2.2.15 cells. (D) qRT-PCR analysis of miR-122 in QSG7701, HepG2, and Huh7 cells transiently transfected with pHBV1.3 for 72 h and the corresponding controls. (E) HepG2.2.15 cells and three different hepatic cell lines transiently transfected with pHBV1.3 were cultured for 72 h; HBV DNA in the supernatants was detected using qRT-PCR with HBV-specific primers. The data represent the mean ± SD. (*p<0.05, **p<0.01, ***p<0.001).

To investigate the effect of HBV on miRNA expression, we performed a miRNA microarray to first survey and compare the miRNA profiles between HepG2 cells and HepG2.2.15 cells. The microarray data were analysed using hierarchical clustering of the log_2_ value and are displayed as a heat map (Figure 1A). Of the 615 identified miRNAs, 62 miRNAs were up-regulated and 151 miRNAs were down-regulated in the HepG2.2.15 compared to HepG2 cells. Among the down-regulated miRNAs, miR-122 was the most dramatically decreased miRNA (Figure 1B). We further confirmed the reduction in miR-122 in HepG2.2.15 cells using qRT-PCR. These results demonstrated that the miR-122 level in HepG2.2.15 cells was dramatically lower compared to HepG2 cells (p = 0.00001, Figure 1C). Moreover, we also found that miR-122 was significantly down-regulated in pHBV1.3-transfected QSG7701 cells compared to controls (p = 0.032), and similar results were obtained when using other cell lines, such as HepG2 (p = 0.0058) and Huh7 cells (p = 0.0046) (Figure 1D). Expression of HBV in these cells was confirmed using PCR (Figure 1E). Here, it was also confirmed that the miR-122 level was reduced in HBV-infected cells.

### HBx is critical for the HBV-induced reduction of miR-122

**Figure 2:**
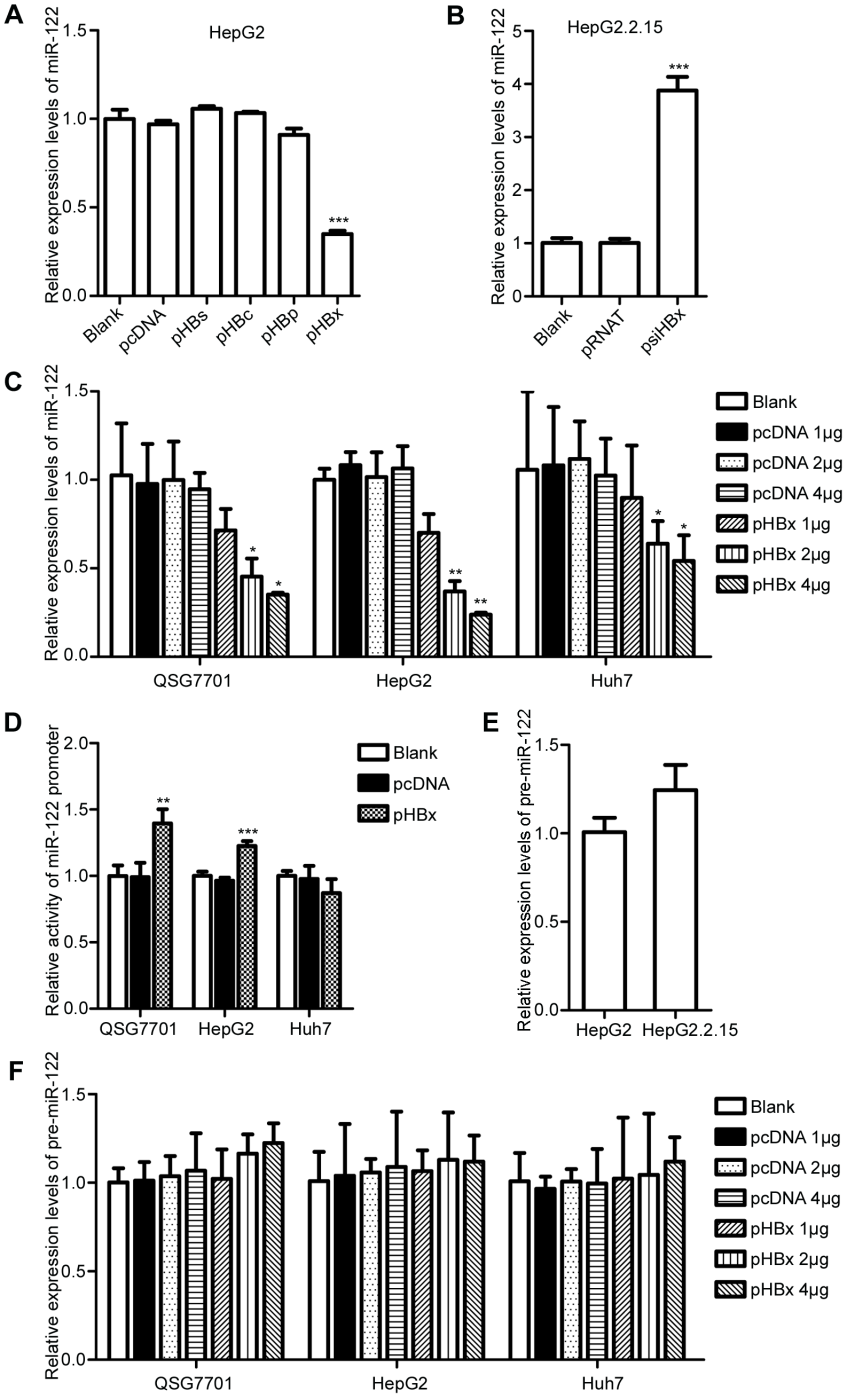
HBx does not reduce miR-122 in hepatic cells via transcriptional regulation. (A) qRT-PCR assay for miR-122 in HBs-, HBc-, HBp-, or HBx-expressing cells. (B) qRT-PCR analysis for miR-122 in HBx-silenced HepG2.2.15. (C) qRT-PCR analysis for miR-122 in three different cell lines (QSG7701, HepG2, Huh7) transfected with pHBx at different doses and the corresponding controls. (D) Dual-luciferase assay of the miR-122 promoter in QSG7701, HepG2, and Huh7 cells transfected with pHBx and controls. (E) qRT-PCR analysis for pre-miR-122 in HepG2 and HepG2.2.15 cells. (F) qRT-PCR analysis for pre-miR-122 in three different cell lines (QSG7701, HepG2, Huh7) transfected with pHBx at different doses and the corresponding controls. The data represent the mean ± SD. (*p<0.05, **p<0.01, ***p<0.001).

We examined the effects of the four HBV proteins on miR-122 by transfecting pHBs, pHBc, pHBp, and pHBx into HepG2 cells. After 72 h of transfection, miR-122 was detected using qRT-PCR. We found that only HBx could reduce miR-122 in hepatic cells (Figure 2A). To further confirm whether miR-122 reduction in HBV-infected cells was related to HBx, psiHBx was transfected into HepG2.2.15 to silence HBx. Interestingly, we found that the inhibitor of HBx could increase the level of miR-122 (p = 0.002, Figure 2B). A similar result was also observed in pHBV1.3-transfected HepG2 cells when HBx was silenced (data not shown). Furthermore, we transfected pHBx into QSG7701, HepG2, and Huh7 cells at different doses and in different cell lines for 72 h. qRT-PCR analysis was performed to detect miR-122 levels. As expected, HBx reduced miR-122 levels in all three cell lines in a dose-dependent manner (Figure 2C). Taken together, these results indicated that HBx, but not other HBV proteins, contributed to the reduction of miR-122 in HBV-infected cells.

### HBV and HBx have no negative effect on miR-122 promoter activity and pre-miR-122 levels

To further investigate the mechanism of HBV-mediated down-regulation of miR-122, we examined the effect of HBx on the activity of the miR-122 promoter. p122-luc was co-transfected with pHBx into QSG7701, HepG2, and Huh7 cells individually, and the dual-luciferase assay was performed. Surprisingly, we found that miR-122 promoter activity was not reduced in HBx-expressing cells compared to controls (Figure 2D). Furthermore, real-time PCR results showed that the precursor miR-122 (pre-miR-122) levels in HBV-infected cells (Figure 2E) and HBx-expressing (Figure 2F) cells were also similar to controls. These results demonstrated that the down-regulation of miR-122 by HBV or HBx was not via transcriptional regulation and that other pathway(s) may exist.

### Gld2 is reduced by HBV and HBx is the only protein encoded by HBV to down-regulate Gld2 gene expression

**Figure 3:**
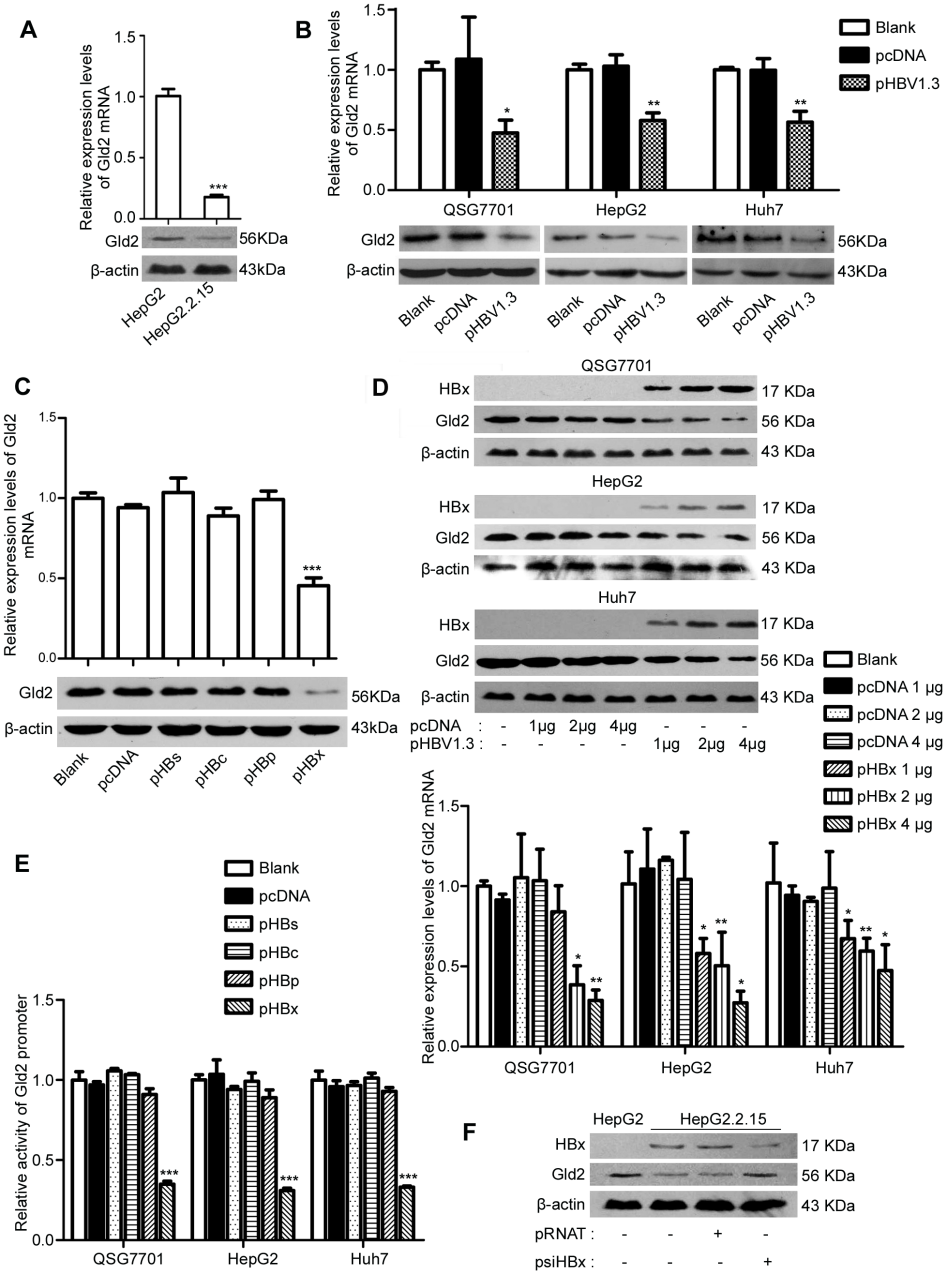
Gld2 was reduced by HBV and HBx. (A) qRT-PCR and western blotting analysis for Gld2 in HepG2 and HepG2.2.15 cells. (B) qRT-PCR and western blotting analysis for Gld2 in three different cell lines after transient transfection with pHBV1.3 and the corresponding controls. (C) qRT-PCR and western blotting analysis for Gld2 in HBs-, HBc-, HBp-, and HBx-expressing HepG2 cells. (D) Western blotting and qRT-PCR analysis for Gld2 in three different cell lines transfected with pHBx at different doses and the corresponding controls. (E) Dual-luciferase assay of Gld2 promoter activity in HBs-, HBc-, HBp-, and HBx-expressing cells and the corresponding controls. (F) Western blotting analysis for Gld2 in HepG2 and HepG2.2.15 cells with or without HBx inhibition. The data represent the mean ± SD. (*p<0.05, **p<0.01, ***p<0.001).

For the specific relationship between Gld2 and miR-122 [21], [22], real-time PCR and western blotting analyses were performed to detect Gld2 mRNA transcript and protein levels in HepG2 and HepG2.2.15 cells. Interestingly, we found that both Gld2 mRNA transcript (p = 0.0002) and protein levels were reduced in HepG2.2.15 cells compared with HepG2 cells (Figure 3A). To further confirm the inhibition of Gld2 induced by HBV, HepG2, QSG7701, and Huh7 cells were transfected with pHBV1.3. As expected, expression of HBV caused the down-regulation of Gld2 in all three cell lines (Figure 3B). Furthermore, pHBs, pHBc, pHBp, and pHBx were transfected into HepG2 cells, and Gld2 was detected after 72 h. These results demonstrated that Gld2 mRNA and protein were reduced only in HBx-expressing cells (Figure 3C). Moreover, a reduction of Gld2 by HBx was observed in three different cell lines and occurred in a dose-dependent manner (Figure 3D). Moreover, we investigated the effects of HBx, HBc, HBp, and HBx on the Gld2 promoter activity in three different cell lines. These results showed that HBx was the only plasmid to reduce Gld2 promoter activity of the four HBV proteins (Figure 3E). In addition, inhibition of HBx in HepG2.2.15 cells increased the expression of Gld2 at both the mRNA (data not shown) and protein levels (Figure 3F). Next, we concluded that HBx, but not HBsAg, HBcAg, or HBp, reduced Gld2 expression in hepatic cells.

### Over-expression of Gld2 rescues the inhibition of miR-122 by HBV or HBx

**Figure 4:**
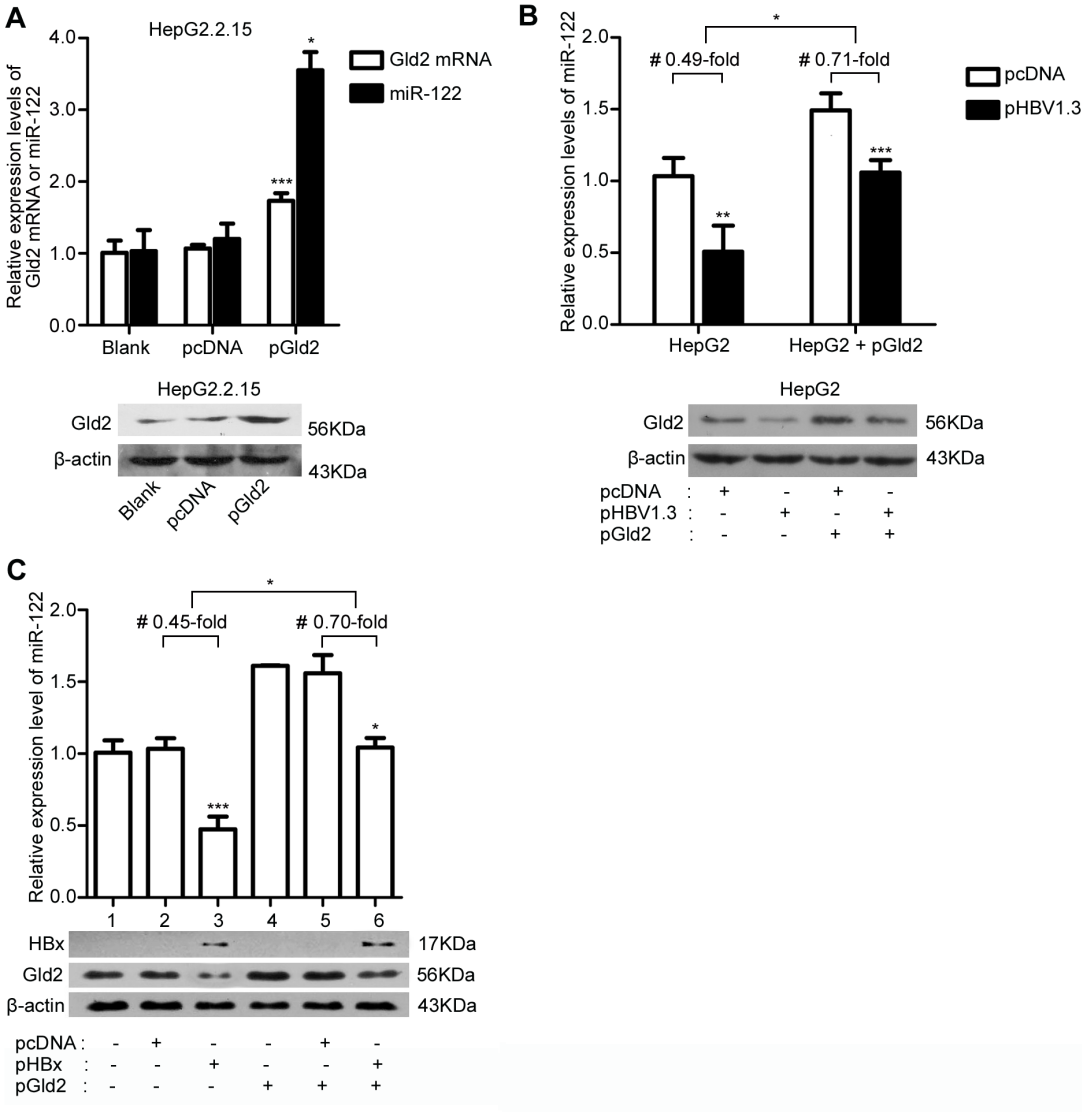
Gld2 contributed to the reduction of miR-122 induced by HBV and HBx. (A) qRT-PCR and western blotting analysis for Gld2 mRNA and miR-122 in HepG2.2.15 with over-expression of Gld2 and controls. (B) qRT-PCR analysis for miR-122 and western blotting analysis for Gld2 in the HepG2 cells co-transfected with pGld2 and pHBV1.3 and the corresponding controls. # the average effects of HBV on the expression of miR-122 in HepG2 cells with or without over-expression of Gld2. (C) qRT-PCR analysis for miR-122 and western blotting analysis for Gld2 in HepG2 cells co-transfected with pGld2 and pHBx and the corresponding controls. # the average effects of HBx on the expression of miR-122 in HepG2 cells with or without over-expression of Gld2. The data represent the mean ± SD. (*p<0.05, **p<0.01, ***p<0.001).

To determine whether the reduction of miR-122 in HBV-infected cells is via the down-regulation of Gld2, we transfected pGld2 into HepG2.2.15 and examined the relative miR-122 levels using real-time PCR. We found that transient expression of Gld2 could increase miR-122 levels in HepG2.2.15 (p = 0.0003, Figure 4A). Moreover, pHBV1.3 and pHBx were transfected into Gld2 over-expressing HepG2 cells and over-expression of Gld2 reduced the negative effect of HBV (Figure 4B) and HBx (Figure 4C) on miR-122. The miR-122 levels in HBV-infected cells with over-expression of Gld2 were about 0.71-fold compared to the controls, while without over-expression of Gld2, the miR-122 levels of HBV-infected cells were 0.49-fold compared to the controls (Figure 4B); the miR-122 levels in HBx-expressing cells with over-expression of Gld2 were about 0.70-fold compared to the controls, while without over-expression of Gld2, the miR-122 levels of HBx-expressing cells were 0.45-fold compared to the controls (Figure 4C). And, the decrease of HBV or HBx's effect on miR-122 induced by over-expression of Gld2 was statistically significant (p<0.05). Finally, we concluded that Gld2 was involved in the reduction of miR-122 in HBV-infected cells and HBx-expressing cells.

## Discussion

In the present study, we explored the mechanism of down-regulation of miR-122 induced by HBV in vitro. First, we confirmed that the miR-122 levels in HepG2.2.15 (a derivative of stable HBV-expressing HepG2 cells) were significantly lower compared to HepG2 cells, which was consistent with the results previously reported by Wu et al. [18]. In addition, Meng et al. found that transfection with the HBV replication plasmid pHBV1.3 significantly decreased miR-122 levels in Huh-7 cells. Furthermore, HBV transgenic mice had lower miR-122 levels in the liver compared to BALB/c mice [17]. Using pHBV1.3, we confirmed the negative effect of HBV on miR-122 in three different cell lines. More importantly, we screened the four HBV proteins and identified the exact protein that reduced miR-122 levels in hepatic cells; HBx was the most effective protein. In this study, we tried to design a siRNA expression vector (psiHBx) to silence HBx in HepG2.2.15 cells. But in fact, the other proteins of HBV, even the HBV DNA in the supernatant of Cell Culture Medium, possessed of different inhibitory action (data not shown). The psiHBx might not specifically inhibit HBx, for the HBx gene overlaps with pregenomic RNA and HBsAg specific RNAs [25]. However, we presented the further evidence that expression of other proteins of HBV have no significant effect on miR-122 (Figure 2A). Taken together, the results from our study and those of others support that HBV and HBx down-regulate miR-122 levels in hepatic cells. However, the route that HBV takes to reduce miR-122 levels remains controversial.

MicroRNA levels are related to transcription, processing, and turnover [26]. Two recent studies have provided two different explanations for the mechanism of miR-122 down-regulation. Meng et al. found that transfection of pHBV1.3 in Huh7 cells could increase miR-122 promoter activity and miR-122 primary transcription (pri-miR-122) rather than decreasing them, which indicated that HBV did not down-regulate miR-122 levels via the transcription pathway [17]. Although the results of Tong et al. indicated that HBx protein could reduce miR-122 promoter activity by binding to PPARγ in both HepG2 and Huh7 cells [18], we found that the activities of the miR-122 promoter varied in different cell lines expressing HBx proteins (increased in HepG2 and QSG7701 cells but non-significantly decreased in Huh7 cells, Fig. 2D). Despite these findings, pre-miR-122 in all of the three cells lines did not decrease when HBV or HBx was expressed. The selected promoter sequences of miR-122 were essentially the same as those of the previous two related studies. However, the different results might be due to the different experimental conditions and/or different cell lines. In previous studies, HBx was found to inhibit the normal function of p53 [27] and p53 could inhibit the expression of hepatocyte nuclear factor 4α (HNF4α) [28], a key regulator of miR-122 expression in the liver [24]. It may implicate that HBx could potentially affect miR-122 promoter activity indirectly and these effects could change under different conditions and/or different cell lines. In the cell lines we used, HepG2 cells were found to express small amounts of wild type p53, whereas Huh7 cells were shown to express mutation p53 [29]. What's more, HNF4α was up-regulated in HBV-infected cells [17]. Though further evidence is needed, it may explain the increased miR-122 promoter activities in QSG7701 and HepG2 cells but not in Huh7 cells. Moreover, we also had the hypothesis that there might be some feedback signal to stimulate the miR-122 promoter when the miR-122 was reduced by HBx, for the positive or negative feedback control signal is often exist to maintain the normal levels of a special object in cells. In addition, HBx was found to down-regulate the Drosha which participate in processing pri-miRNA to release pre-miRNA [30]. It may explain the increased miR-122 promoter activities resulted in the similar pre-miR-122 levels. Thus, the relationship between HBx and miR-122 may be more complicated than we have known. Since the transcription pathway cannot always explain the low miR-122 levels in HBx-expressing cells, there must be other pathway(s) related to the down-regulation of miR-122 induced by HBx.

Gld2 has been shown to have important effects on the stability of many miRNAs, including miR-122, via catalysed 3′ monoadenylation [21]–[23]. A decrease in Gld2 levels could cause a reduction in mature miR-122 levels but not pre-miR-122 levels [22]. Here, we found that HepG2.2.15 cells had lower Gld2 mRNA and protein levels compared to HepG2 cells (Figure 3A), which suggested that the change in Gld2 was positively correlated with miR-122. Moreover, transfection of HBx in vitro also resulted in a reduction in Gld2 expression both at the mRNA and protein levels (Figure 3D). Furthermore, inhibition of HBx in HBV-infected cells could increase miR-122 and Gld2 levels (Figure 3F). In addition, the expression of Gld2 could abolish the reduction of miR-122 induced by HBV and HBx (Figure 4B, 4C). These findings indicated that down-regulation of miR-122 by HBV or HBx might be due to a reduction in Gld2. However, when we transfected the pGld2 to over-expressing Gld2 in hepatic cells, the endogenous Gld2 was still the main parts of the total Gld2 (more than 60%, Figure 4B and 4C). Thus, the effect of HBV or HBx to endogenous Gld2 was hard to be covered up by the exogenous over-expression of Gld2 and it may be partly explain the Gld2 over expression could not completely rescue the down-regulation effect of HBV infection and HBx expression to miR-122. Of course, other path ways to reduce the miR-122 by HBV cannot be denied. Furthermore, we found that the activity of the Gld2 promoter was reduced in all three cell lines when the cells were transfected with HBx-expressing plasmids. A review has pointed out that HBx does not directly bind DNA, and regulates transcription by direct interaction with nuclear transcription components or activation of cytosolic signal transduction pathways [25]. Hence, further studies are needed to investigate the precise transcription factor that induced the relationship between HBx and Gld2. Furthermore, HBx/Gld2/miR-122 may be one important pathway for hepatocarcinogenesis, representing a correlation between miR-122 and liver cancer [6]–[9].

Interestingly, according to a study completed by D'Ambrogio et al., there are approximately 50 miRNAs that may be monoadenylated by Gld2 [22]. Thus, HBx may affect other miRNAs via the down-regulation of Gld2 expression. In our study, four of five miRNAs (let-7i, let-7d, miR-145, miR-22, and miR-122), which are stabilised by Gld2 [22], were analysed in our miRNA microarray results. In addition, all of the miRNAs were down-regulated in HepG2.2.15 compared to HepG2 cells (data not shown). However, real-time PCR or northern blotting analyses should be performed to confirm these findings. In addition, information indicating that Gld2 may be an important factor for the effect of HBV on miRNAs is needed. There may be several regulatory mechanisms that coexist to regulate miR-122 levels in HBV-infected cells.

Taken together, we found that Gld2 is down-regulated by HBx, which results in the reduction of miR-122 levels in HBV-infected cells. This process is post-transcriptionally regulated. Thus, this study provides a new mechanism to explain the effect of HBV on miRNA expression.

## References

[1] Chang J, Nicolas E, Marks D, Sander C, Lerro A, et al (2004) miR-122,a Mammalian liver-specific microRNA,is Processed from hcr mRNA and May Downregulate the High Affinity Cationic Amino Acid Transporter CAT-1. RNA Biol 1: 106–113. 17179747 10.4161/rna.1.2.1066

[2] Hu J, Xu Y, Hao J, Wang S, Li C, et al (2012) MiR-122 in hepatic function and liver diseases. Protein Cell 3: 364–371 doi:10.1007/s13238-012-2036-3 22610888 10.1007/s13238-012-2036-3 PMC4875471

[3] Esau C, Davis S, Murray SF, Yu XX, Pandey SK, et al (2006) miR-122 regulation of lipid metabolism revealed by in vivo antisense targeting. Cell Metab 3: 87–98. 16459310 10.1016/j.cmet.2006.01.005

[4] Wen J, Friedman JR (2012) miR-122 regulates hepatic lipid metabolism and tumor suppression. J Clin Invest 122: 2773–2776. 22820284 10.1172/JCI63966 PMC3408753

[5] Hsu S, Wang B, Kota J, Yu J, Costinean S, et al (2012) Essential metabolic, anti-inflammatory, and anti-tumorignic functions of miR-122 in liver. J Clin Invest 122: 2871–2883. 22820288 10.1172/JCI63539 PMC3408748

[6] Lin CJF, Gong HY, Tseng HC, Wang WL, Wu JL (2008) miR-122 targets an anti-apoptotic gene, Bcl-w, in human hepatocellular carcinoma cell lines. Biochem Biophys Res Commun 375: 315–320. 18692484 10.1016/j.bbrc.2008.07.154

[7] Gramantieri L, Ferracin M, Fornari F, Veronese A, Sabbioni S, et al (2007) Cyclin G1 is a target of miR-122a, a microRNA frequently down-regulated in human hepatocellular carcinoma. Cancer Res 67: 6092–6099. 17616664 10.1158/0008-5472.CAN-06-4607

[8] Bai S, Nasser MW, Wang B, Hsu S-H, Datta J, et al (2009) MicroRNA-122 inhibits tumorigenic properties of hepatocellular carcinoma cells and sensitizes these cells to sorafenib. J Biol Chem 284: 32015–32027. 19726678 10.1074/jbc.M109.016774 PMC2797273

[9] Kutay H, Bai S, Datta J, Motiwala T, Pogribny I, et al (2011) Downregulation of miR-122 in the Rodent and Human Hepatocellular Carcinomas. J Cell Biochem 99: 671–678 doi:10.1002/jcb.20982.Downregulation 10.1002/jcb.20982 PMC3033198 16924677

[10] Coulouarn C, Factor VM, Andersen JB, Durkin ME, Thorgeirsson SS (2009) Loss of miR-122 expression in liver cancer correlates with suppression of the hepatic phenotype and gain of metastatic properties. Oncogene 28: 3526–3536. 19617899 10.1038/onc.2009.211 PMC3492882

[11] Tsai WC, Hsu PWC, Lai TC, Chau GY, Lin CW, et al (2009) MicroRNA-122, a tumor suppressor microRNA that regulates intrahepatic metastasis of hepatocellular carcinoma. Hepatology 49: 1571–1582. 19296470 10.1002/hep.22806

[12] Ma L, Liu J, Shen J, Liu L, Wu J, et al (2010) Expression of miR-122 mediated by adenoviral vector induces apoptosis and cell cycle arrest of cancer cells. Cancer Biol Ther 9: 554–561. 20150764 10.4161/cbt.9.7.11267

[13] Chang J, Guo JT, Jiang D, Guo H, Taylor JM, et al (2008) Liver-specific microRNA miR-122 enhances the replication of hepatitis C virus in nonhepatic cells. J Virol 82: 8215–8223. 18550664 10.1128/JVI.02575-07 PMC2519557

[14] Janssen HL a, Reesink HW, Lawitz EJ, Zeuzem S, Rodriguez-Torres M, et al (2013) Treatment of HCV infection by targeting microRNA. N Engl J Med 368: 1685–1694. 23534542 10.1056/NEJMoa1209026

[15] Wang S, Qiu L, Yan X, Jin W, Wang Y, et al (2012) Loss of microRNA 122 expression in patients with hepatitis B enhances hepatitis B virus replication through cyclin G(1) -modulated P53 activity. Hepatology 55: 730–741. 22105316 10.1002/hep.24809

[16] Waidmann O, Bihrer V, Pleli T, Farnik H, Berger a, et al (2012) Serum microRNA-122 levels in different groups of patients with chronic hepatitis B virus infection. J Viral Hepat 19: e58–65. 22239527 10.1111/j.1365-2893.2011.01536.x

[17] Li C, Wang Y, Wang S, Wu B, Hao J, et al (2013) Hepatitis B virus mRNA-mediated miR-122 inhibition upregulates PTTG1-binding protein, which promotes hepatocellular carcinoma tumor growth and cell invasion. J Virol 87: 2193–2205. 23221562 10.1128/JVI.02831-12 PMC3571498

[18] Song K, Han C, Zhang J, Lu D, Dash S (2013) Epigenetic regulation of miR-122 by PPARγ and hepatitis B virus X protein in hepatocellular carcinoma cells. Hepatology 58: 1681–1692. 23703729 10.1002/hep.26514 PMC3773012

[19] Kwak JE, Wang L, Ballantyne S, Kimble J, Wickens M (2004) Mammalian GLD-2 homologs are poly(A) polymerases. Proc Natl Acad Sci U S A 101: 4407–4412. 15070731 10.1073/pnas.0400779101 PMC384760

[20] Glahder J, Norrild B (2011) Involvement of hGLD-2 in cytoplasmic polyadenylation of human p53 mRNA. APMIS 199: 769–775. 10.1111/j.1600-0463.2011.02804.x 21995630

[21] Burns DM, D'Ambrogio A, Nottrott S, Richter JD (2011) CPEB and two poly(A) polymerases control miR-122 stability and p53 mRNA translation. Nature 473: 105–108. 21478871 10.1038/nature09908 PMC3088779

[22] D'Ambrogio A, Gu W, Udagawa T, Mello CC, Richter JD (2012) Specific miRNA stabilization by Gld2-catalyzed monoadenylation. Cell Rep 2: 1537–1545. 23200856 10.1016/j.celrep.2012.10.023 PMC3534910

[23] Katoh T, Sakaguchi Y, Miyauchi K, Suzuki T, Kashiwabara SI, et al (2009) Selective stabilization of mammalian microRNAs by 3′ adenylation mediated by the cytoplasmic poly(A) polymerase GLD-2. Genes Dev 23: 433–438. 19240131 10.1101/gad.1761509 PMC2648654

[24] Li ZY, Xi Y, Zhu WN, Zeng C, Zhang ZQ, et al (2011) Positive regulation of hepatic miR-122 expression by HNF4α. J Hepatol 55: 602–611. 21241755 10.1016/j.jhep.2010.12.023

[25] Bouchard MJ, Schneider RJ, Bouchard MJ, Schneider RJ (2004) The Enigmatic X Gene of Hepatitis B Virus. J Virol 78: 12725–12734. 15542625 10.1128/JVI.78.23.12725-12734.2004 PMC524990

[26] JM, Newman M, Parker JS, Morin Kensicki EM, Wright T, et al (2006) Extensive post-transcriptional regulation of microRNAs and its implications for cancer. Genes Dev 20: 2202–2207. 16882971 10.1101/gad.1444406 PMC1553203

[27] Murakami S (1999) Hepatitis B virus X protein: structure, function and biology. Intervirology 42: 81–99. 10516464 10.1159/000024969

[28] Maeda Y, Hwang Verslues WW, Wei G, Fukazawa T, Durbin ML, et al (2006) Tumour suppressor p53 down-regulates the expression of the human hepatocyte nuclear factor 4alpha (HNF4alpha) gene. Biochem J 400: 303–313. 16895524 10.1042/BJ20060614 PMC1652821

[29] Müller BM, Wilder S, Bannasch D, Israeli D, Lehlbach K, et al (1998) p53 Activates the CD95(APO-1/Fas) Gene in Response to DNA Damage by Anticancer Drugs. J Exp Med 188: 2033–2045. 9841917 10.1084/jem.188.11.2033 PMC2212386

[30] Ren M, Qin D, Li K, Qu J, Wang L, et al (2012) Correlation between hepatitis B virus protein and microRNA processor Drosha in cells expressing HBV. Antiviral Res 94: 225–231. 22554933 10.1016/j.antiviral.2012.04.004

